# Modeling and Validation of Vertical Direction Force Estimation with a Three-Dimensional Force Measurement Instrument Based on a Zero-Compliance Mechanism

**DOI:** 10.3390/s19040799

**Published:** 2019-02-15

**Authors:** Md Helal An Nahiyan, Takeshi Mizuno, Masaya Takasaki, Yuji Ishino, Masayuki Hara, Daisuke Yamaguchi

**Affiliations:** Department of Mechanical Engineering, Saitama University, Saitama 338-8570, Japan; mizar@mech.saitama-u.ac.jp (T.M.); masaya@mech.saitama-u.ac.jp (M.T.); yishino@mech.saitama-u.ac.jp (Y.I.); masayuki@mech.saitama-u.ac.jp (M.H.); yamaguchi14@mech.saitama-u.ac.jp (D.Y.)

**Keywords:** zero-compliance, force measurement, electromagnetic force, magnetic balance, series suspension

## Abstract

The principle of a zero-compliance mechanism was used to develop a three-dimensional force measurement instrument. In each axis, the point of force is suspended by a zero-compliance mechanism. A vertical axis force estimation operation imitates the structure of a double series magnetic suspension system. An electromagnet directly controls the movement of the first suspended object (floator), which is denoted as a detection point, and indirectly controls the motion of the second floator, which is denoted as a point of force. Indirect control of the point of force is executed by the attractive force of a permanent magnet that is fixed to the bottom part of the detection point. To achieve zero-compliance, a Proportional-Integral-Derivative (PID) control is applied to the point of force, and to make the system stable, a Proportional-Derivative (PD) control is also applied to the detection point. In such suspension conditions, when force is exerted on the point of force, the displacement of the second floator is regulated to maintain its primary position while the detection point displaces in proportion to the applied force. Thus, a zero-compliance condition is maintained at the point of force, and the external force is measured from the linear displacement of the detection point. To restrict the motions of the detection point and the point of force in translation only, they are supported with leaf springs. This paper presents the modeling of the vertical direction force measurement operation of the developed three-axis force estimation instrument, and the theoretical analyses were validated by experiments of force measurement in both the millinewton and micronewton ranges.

## 1. Introduction

Force measurement is immensely important in numerous scientific research and industrial applications, especially in the development of new materials, where the assembly of molecules is essential. Measuring small force is inevitable in modern micro-assembly and micromanipulation. There are several force measurement techniques that have already been developed [1,2,3]. Some research has focused on measuring small force by using strain gauge-based force sensors [4,5,6], piezoelectric actuators [7,8], Micro-Electro-Mechanical System (MEMS) sensors [9,10], optical force sensors [11] and so on. For measuring microforce in diversified fields from biological research to material sciences, an atomic force microscope (AFM) cantilever is a widely used tool [12,13]. Similarly to other deflection methods [14,15], an atomic force microscope cantilever estimates the force from the deflection of the cantilever. In these systems, for measuring small forces with higher resolution, the stiffness should be sufficiently low. However, the low stiffness causes a large change of gap between the source of force and the point of force. In contrast, without displacing the point of force, the applied force is estimated from the control current of the magnetic suspension balance [16,17]. However, the noise in the control current signal disturbs precise measurement when the applied force is small. To solve these problems, force estimation using zero-compliance has been proposed [18]. It ensures high sensitivity in force estimation and infinite stiffness at the point of force. 

Several systems have already been developed for zero-compliance force measurements using a double series magnetic suspension system. The first device focused on vertical axis force estimation [19], in which an electromagnet controls the motion of both the point of force and the detection point, with a permanent magnet installed under the detection point. The detection point is guided by leaf springs, and the point of force is suspended without any contact. A similar structure followed in the development of a triaxial force measurement apparatus [20]. Both devices have been used to measure force in the micronewton (μN) range. However, due to complete suspension, the point of force fluctuates even in the zero-compliance condition, which disturbs the accurate estimation of force. Another single direction force measurement device was constructed by suspending both floators through leaf springs [21]. This device solved the problem of fluctuation of the point of force. However, measurement capability is restricted to a single direction only, and the force measurement is in the millinewton (mN) range. 

In response to the above-mentioned problems, a three-dimensional force measurement instrument was developed to obtain a stable zero-compliance condition at the point of force and to measure force in the mN range [22,23]. To avoid fluctuations, both the point of force and the detection point are suspended with leaf springs. In addition, electromagnets are replaced by voice coil motors to regulate the motions of the point of force and the detection point in the lateral directions to avoid the nonlinear characteristics of the permanent magnet. In this work, the developed device was modified for force measurement in the μN range by using high-resolution laser sensors. The laser sensors estimate the displacements of the point of force and the detection point in the micrometer (μm) range. A force of an μN amount is exerted on the point of force with an additional voice coil motor. The capability of the developed instrument for stable zero-compliance force measurement was derived through mathematical modeling of the vertical direction force measurement operation. Then, the outcome of the modeling was validated with experimental results of force measurement in the mN and μN ranges. Dynamic force measurements were also demonstrated to confirm the stable zero-compliance condition at the point of force for dynamic forces. Dynamic force was estimated up to 0.5 Hz frequency, whereas the previously developed device [21] is restricted to 0.1 Hz.

## 2. Materials and Methods

### 2.1. Principle of Zero-Compliance

**Figure 1:**
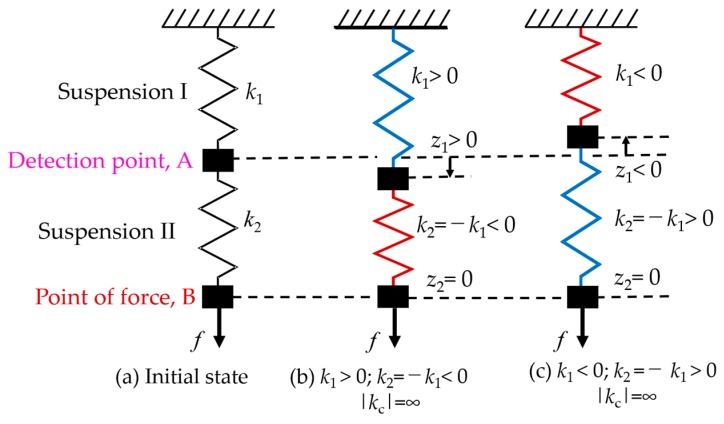
Zero-compliance by using series suspension [23]. (**a**) Initial state of the suspension; (**b**) Positive stiffness is set for Suspension I and negative stiffness is set for Suspension II; (**c**) Negative stiffness is set for Suspension I and positive stiffness is set for Suspension II.

The basic zero-compliance force measurement principle is presented in Figure 1 [19]. There are two suspensions (Suspension I and Suspension II) connected in a series, where the connection point of the two suspensions A is the detection point, and the free end B is the point of force. The combined stiffness (*k*_c_) can be represented as:

(1) $$k_{c}=\frac{k_{1}k_{2}}{k_{1}+k_{2}},$$

where *k*_1_ and *k*_2_ are the stiffness of Suspension I and Suspension II, respectively. Equation (1) represents that the combined stiffness is smaller than the individual stiffness of Suspension I and Suspension II when normal springs (*k*_i_ > 0) are connected in a series. In contrast, the stiffness of the suspensions is selected, as displayed by Figure 1b,c:

(2) $$(b)\;k_{1}>0\;\mathrm{and}\;k_{2}=-k_{1}<0$$

or

(3) $$(c)\;k_{1}<0\;\mathrm{and}\;k_{2}=-k_{1}>0.$$

The resultant stiffness becomes infinite. This means the point of force maintains its original position even when force is exerted, and the detection point displaces in proportion to the applied force. Moreover, as presented by Figure 1b, when positive stiffness is set to Suspension I, the detection point moves in the same direction as the applied force: In contrast, the detection point displaces in the direction opposite to the applied force when Suspension II has positive stiffness, as shown by Figure 1c:

(4) $$\left(b\right)\;z_{1}=\frac{f}{k_{1}}=-\frac{f}{k_{2}}>0$$

or

(5) $$\left(c\right)\;z_{1}=\frac{f}{k_{1}}=-\frac{f}{k_{2}}<0.$$

In both configurations, therefore, applied force can be estimated from the displacement of the detection point.

### 2.2. Force Measurement Using a Double Series Magnetic Suspension System

**Figure 2:**
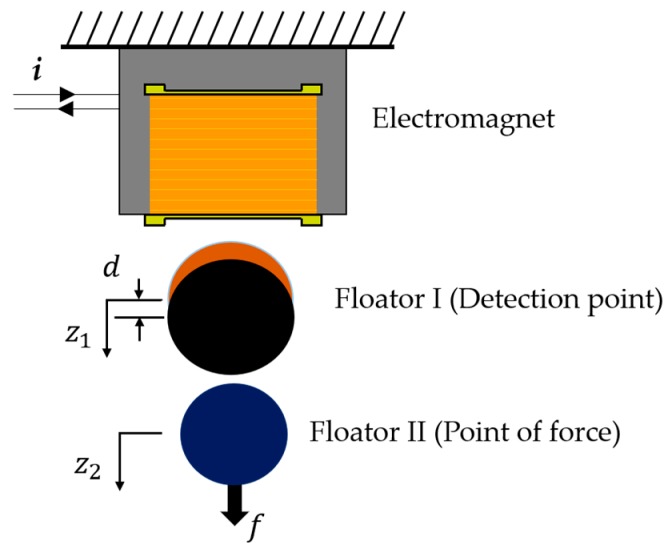
Force measurement with double series magnetic suspension system.

A zero-compliance condition is achievable by using a double series magnetic suspension system. As shown by Figure 2, there are two floaters suspended in series by an electromagnet. The electromagnet directly controls the movement of Floator I (detection point). A permanent magnet is attached to the bottom of Floator I and controls the motion of Floator II (point of force). To achieve stable suspension and zero-compliance, a PID control to the point of force and a PD control to the detection point are applied. If force is applied in such suspension conditions, Floator I displaces downward to balance the applied force on Floator II. Therefore, the gap between Floator I and Floator II is reduced and Floator II maintains its position.

## 3. Results

**Figure 3:**
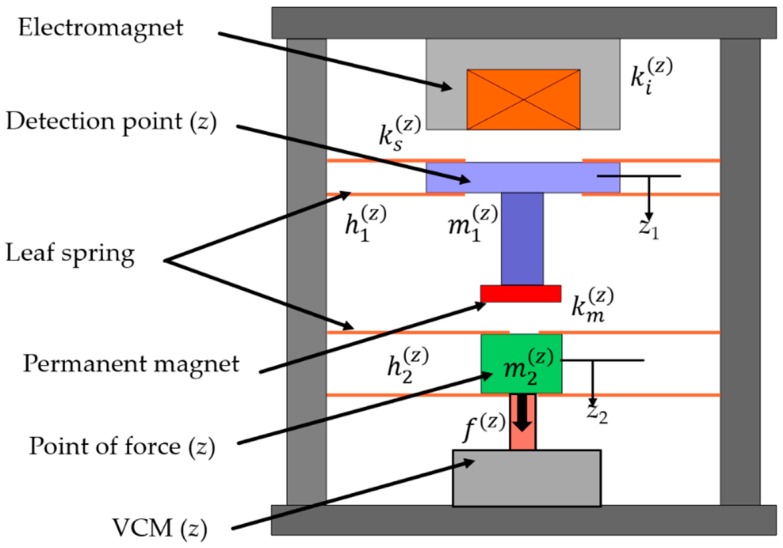
View of the vertical axis force estimation operation [23].

By following a similar mechanism of a double series magnetic suspension system, vertical axis force is estimated. In contrast with the previous apparatus [19,20], both the point of force and the detection point are supported with leaf springs here, as shown in Figure 3. Thus, the equations of motion become:

(6) $$m_{1}^{\left(z\right)}{\ddot{z}}_{1}\left(t\right)=-k_{s1}^{\left(z\right)}z_{1}\left(t\right)+k_{m}^{\left(z\right)}\left(z_{1}\left(t\right)-z_{1}\left(t\right)\right)-k_{i}^{\left(z\right)}i^{\left(z\right)}\left(t\right),$$

(7) $$m_{2}^{\left(z\right)}{\ddot{z}}_{2}\left(t\right)=-h_{2}^{\left(z\right)}z_{2}\left(t\right)-k_{m}^{\left(z\right)}\left(z_{1}\left(t\right)-z_{1}\left(t\right)\right)+f^{\left(z\right)}\left(t\right),$$

where $z_{1}$ and $z_{2}$ are displacements of the detection point and the point of force; $m_{1}^{\left(z\right)}$ and $m_{2}^{\left(z\right)}$ are masses of the detection point (*z*) and the point of force (*z*); $h_{1}^{\left(z\right)}$ and $h_{2}^{\left(z\right)}$ are the stiffnesses of the leaf springs connected to the detection point and the point of force; $k_{i}^{\left(z\right)}$ is the electromagnet current force coefficient;$\;k_{s}^{\left(z\right)}$ is the electromagnet gap force factor; $i^{\left(z\right)}$ is the electromagnet control current; $k_{m}^{\left(z\right)}$ is the permanent magnet gap force factor; $k_{s1}^{\left(z\right)}$ is the combination of the gap force factor of the electromagnet and the leaf spring stiffness connected to the detection point; and $f^{\left(z\right)}$ is the external force applied to the point of force. 

Similarly to the double series magnetic suspension, in the vertical axis force estimation operation, a PD control is applied to the detection point and a PID control is applied to the point of force. The control current of the electromagnet can be represented as

(8) $$I^{\left(z\right)}\left(s\right)=\left(p_{d}^{\left(z\right)}\;+{\;\mathrm{sp}}_{v}^{\left(z\right)}\right){\;Z}_{1}\left(s\right)\;-\;\left(q_{d}^{\left(z\right)}+{\mathrm{sq}}_{v}^{\left(z\right)}+\frac{q_{I}^{\left(z\right)}}{s}\right){\;Z}_{2}\left(s\right),$$

where $p_{d}^{\left(z\right)}$ and $p_{v}^{\left(z\right)}$ are proportional and derivative gains of the PD controller; and $q_{d}^{\left(z\right)},\;q_{v}^{\left(z\right)}$, and $q_{I}^{\left(z\right)}$ are proportional, derivative, and integral gains of the PID controller.

**Figure 4:**
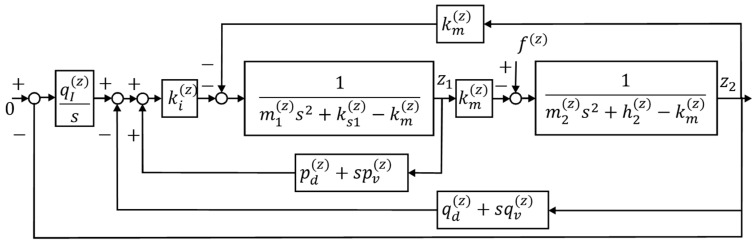
Block diagram of the vertical direction force measurement [23].

A control system block diagram of the vertical axis force estimation operation is presented in Figure 4.

Laplace transform of Equations (6) and (7) give

(9) $$m_{1}^{\left(z\right)}s^{2}Z_{1}\left(s\right)=\;-k_{s1}^{\left(z\right)}Z_{1}\left(s\right)+k_{m}^{\left(z\right)}\left(Z_{1}\left(s\right)-Z_{2}\left(s\right)\right)-k_{i}^{\left(z\right)}I^{\left(z\right)}\left(s\right),$$

(10) $$m_{2}^{\left(z\right)}s^{2}Z_{2}\left(s\right)=\;-h_{2}^{\left(z\right)}Z_{2}\left(s\right)-k_{m}^{\left(z\right)}\left(Z_{1}\left(s\right)-Z_{2}\left(s\right)\right)+F^{\left(z\right)}\left(s\right).$$

From Equations (9) and (10), the relationship between $Z_{1}$ and $Z_{2}$ can be written as

(11) $$\frac{Z_{2}}{Z_{1}}=\frac{m_{1}^{\left(z\right)}s^{2}+k_{s1}^{\left(z\right)}-k_{m}^{\left(z\right)}+k_{i}^{\left(z\right)}\left(p_{d}^{\left(z\right)}+{\mathrm{sp}}_{v}^{\left(z\right)}\right)}{k_{i}^{\left(z\right)}\left(q_{d}^{\left(z\right)}+{\mathrm{sq}}_{v}^{\left(z\right)}+\frac{q_{I}^{\left(z\right)}}{s}\right)-k_{m}^{\left(z\right)}}.$$

Thus, the transfer function equations of $Z_{1}$ and $Z_{2}$ are given by

(12) $$Z_{1}\left(s\right)=G_{1F}\left(s\right)\;F^{\left(z\right)}\left(s\right),$$

(13) $$Z_{2}\left(s\right)=G_{2F}\left(s\right){\;F}^{\left(z\right)}\left(s\right),$$

where

(14) $$G_{1F}\left(s\right)=\frac{\left\{\frac{k_{i}^{\left(z\right)}q_{v}^{\left(z\right)}}{m_{1}^{\left(z\right)}m_{2}^{\left(z\right)}}s^{2}+\frac{\left(k_{i}^{\left(z\right)}q_{d}^{\left(z\right)}-k_{m}^{\left(z\right)}\right)}{m_{1}^{\left(z\right)}m_{2}^{\left(z\right)}}s+\frac{k_{i}^{\left(z\right)}q_{I}^{\left(z\right)}}{m_{1}^{\left(z\right)}m_{2}^{\left(z\right)}}\right\}}{D^{\left(z\right)}\left(s\right)},$$

(15) $$G_{2F}\left(s\right)=\frac{\left\{\frac{1}{m_{2}^{\left(z\right)}}s^{3}+\frac{k_{i}^{\left(z\right)}p_{v}^{\left(z\right)}}{m_{1}^{\left(z\right)}m_{2}^{\left(z\right)}}s^{2}+\frac{\left(k_{s1}^{\left(z\right)}-k_{m}^{\left(z\right)}+k_{i}^{\left(z\right)}p_{d}^{\left(z\right)}\right)}{m_{1}^{\left(z\right)}m_{2}^{\left(z\right)}}s\right\}}{D^{\left(z\right)}\left(s\right)},$$

(16) $$\begin{array}{c} D^{\left(z\right)}\left(s\right)=s^{5}+\frac{k_{i}^{\left(z\right)}p_{v}^{\left(z\right)}}{m_{1}^{\left(z\right)}}s^{4}+\left\{\frac{\left(h_{2}^{\left(z\right)}-k_{m}^{\left(z\right)}\right)}{m_{2}^{\left(z\right)}}+\frac{\left(k_{s1}^{\left(z\right)}-k_{m}^{\left(z\right)}+k_{i}^{\left(z\right)}p_{d}^{\left(z\right)}\right)}{m_{1}^{\left(z\right)}}\right\}s^{3}+\\ \frac{\left\{k_{i}^{\left(z\right)}p_{v}^{\left(z\right)}\left(h_{2}^{\left(z\right)}-k_{m}^{\left(z\right)}\right)+k_{m}^{\left(z\right)}k_{i}^{\left(z\right)}q_{v}^{\left(z\right)}\right\}}{m_{1}^{\left(z\right)}m_{2}^{\left(z\right)}}s^{2}+\frac{\left\{\begin{array}{c} \left(h_{2}^{\left(z\right)}-k_{m}^{\left(z\right)}\right)\left(k_{s1}^{\left(z\right)}+k_{i}^{\left(z\right)}p_{d}^{\left(z\right)}\right)\\ +k_{m}^{\left(z\right)}\left(k_{i}^{\left(z\right)}q_{d}^{\left(z\right)}-h_{2}^{\left(z\right)}\right) \end{array}\right\}}{m_{1}^{\left(z\right)}m_{2}^{\left(z\right)}}s+\frac{k_{m}^{\left(z\right)}k_{i}^{\left(z\right)}q_{I}^{\left(z\right)}}{m_{1}^{\left(z\right)}m_{2}^{\left(z\right)}}. \end{array}$$

From Equation (16), the poles of the system are assigned arbitrarily by selecting the feedback gains $p_{d}^{\left(z\right)}$, $p_{v}^{\left(z\right)}$, $q_{d}^{\left(z\right)}$, $q_{v}^{\left(z\right)}$, and $q_{I}^{\left(z\right)}$.

The Butterworth characteristics polynomial of a fifth-order system can be expressed as follows:

(17) $$D^{\left(z\right)}\left(s\right)=s^{5}+\beta_{4}s^{4}+\beta_{3}{\;s}^{3}+\beta_{2}{\;s}^{2}+\beta_{1}s+\beta_{0},$$

where $\beta_{4}=3.236\;\omega_{n}$, $\beta_{3}=5.236\;\omega_{n}^{2}$, $\beta_{2}=5.236\;\omega_{n}^{3}$, $\beta_{1}=3.236{\;\omega }_{n}^{4}$, $\beta_{0}=\omega_{n}^{5}$.

By equating Equations (16) and (17) for the coefficients of $s^{4},\;\;s^{3},\;\;s^{2},\;\;s,$ and $s^{0}$, the expressions of $p_{d}^{\left(z\right)}$, $p_{v}^{\left(z\right)}$, $q_{d}^{\left(z\right)}$, $q_{v}^{\left(z\right)}$, and $q_{I}^{\left(z\right)}$ can be represented by

(18) $$p_{v}^{\left(z\right)}=\frac{m_{1}^{\left(z\right)}}{k_{i}^{\left(z\right)}}\beta_{4},$$

(19) $$p_{d}^{\left(z\right)}=\beta_{3}\frac{m_{1}^{\left(z\right)}}{k_{i}^{\left(z\right)}}-\frac{\left(h_{2}^{\left(z\right)}-k_{m}^{\left(z\right)}\right)}{m_{2}^{\left(z\right)}}\frac{m_{1}^{\left(z\right)}}{k_{i}^{\left(z\right)}}-\frac{\left(k_{s1}^{\left(z\right)}-k_{m}^{\left(z\right)}\right)}{k_{i}^{\left(z\right)}},$$

(20) $$q_{v}^{\left(z\right)}=\frac{\beta_{2}m_{1}^{\left(z\right)}m_{2}^{\left(z\right)}-\beta_{4}m_{1}^{\left(z\right)}\left(h_{2}^{\left(z\right)}-k_{m}^{\left(z\right)}\right)}{k_{m}^{\left(z\right)}k_{i}^{\left(z\right)}},$$

(21) $$q_{d}^{\left(z\right)}=\frac{\left(h_{2}^{\left(z\right)}-k_{m}^{\left(z\right)}\right)\left\{\beta_{1}m_{1}^{\left(z\right)}m_{2}^{\left(z\right)}-\beta_{3}m_{1}^{\left(z\right)}-\frac{\left(h_{2}^{\left(z\right)}-k_{m}^{\left(z\right)}\right)m_{1}^{\left(z\right)}}{m_{2}^{\left(z\right)}}+k_{m}^{\left(z\right)}\right\}+h_{2}^{\left(z\right)}k_{m}^{\left(z\right)}}{k_{i}^{\left(z\right)}},$$

(22) $$q_{I}^{\left(z\right)}=\frac{m_{1}^{\left(z\right)}m_{2}^{\left(z\right)}}{k_{m}^{\left(z\right)}k_{i}^{\left(z\right)}}\beta_{0}.$$

Therefore, arbitrary pole placement and achieving zero-compliance are possible by determining the feedback gains $p_{d}^{\left(z\right)}$, $p_{v}^{\left(z\right)}$, $q_{d}^{\left(z\right)}$, $q_{v}^{\left(z\right)}$, and $q_{I}^{\left(z\right)}$. The feedback gains can be computed from Equations (18) to (22) for various $f_{n}\left(=\frac{\omega_{n}}{2\pi }\right)$, as shown by Table 1.

**Figure 5:**
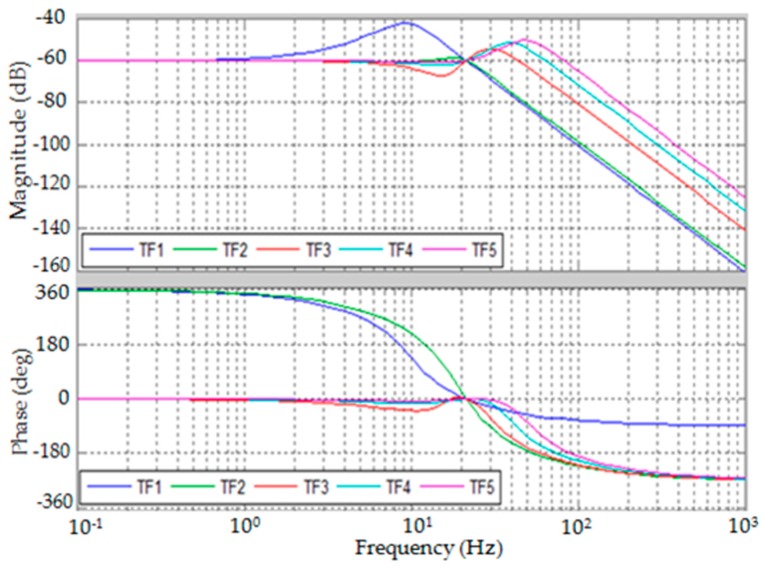
Bode plot of $G_{1F}\left(s\right)$.

**Figure 6:**
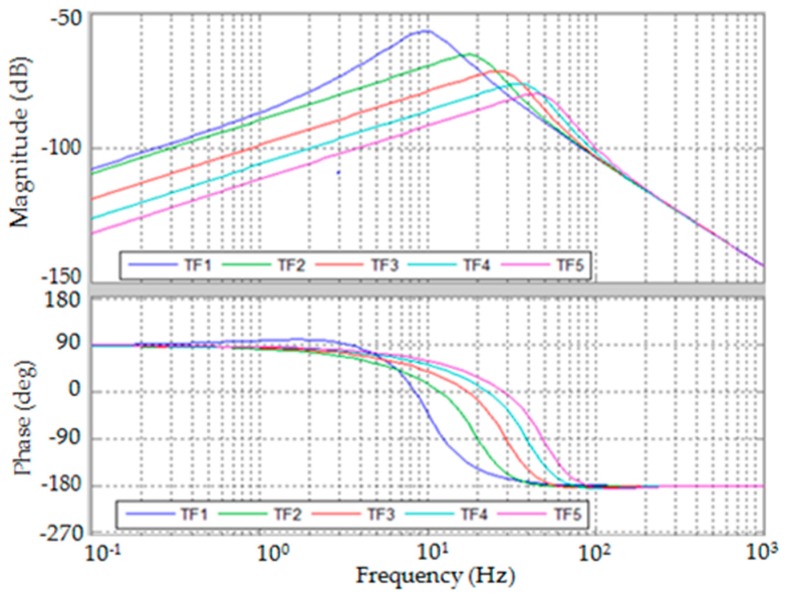
Plot of $G_{2F}\left(s\right)$.

The bode plots of $G_{1F}\left(s\right)$ and $G_{2F}\left(s\right)$ are presented by Figure 5 and Figure 6. The bandwidth of dynamic force measurement can be estimated from the bode plots by selecting the control parameters. However, in actual cases, the control parameters may be limited for structural stability.

Constant force is exerted on the point of force, which is represented as

(23) $$F^{\left(z\right)}\left(s\right)=\frac{F_{0}^{\left(z\right)}}{s}.$$

The steady state displacements are given by

(24) $$z_{1}\left(\infty \right)=\underset{t\to \infty }{\lim}z_{1}\left(t\right)=\underset{s\to 0}{\lim}sZ_{1}\left(s\right)=\frac{F_{0}^{\left(z\right)}}{k_{m}^{\left(z\right)}},$$

(25) $$z_{2}\left(\infty \right)=\underset{t\to \infty }{\lim}z_{2}\left(t\right)=\underset{s\to 0}{\lim}sZ_{2}\left(s\right)=0.$$

Equation (23) demonstrates that the position of the point of force is kept invariant as if it is suspended by an infinite-stiffness spring. Therefore, the distance between the source of force and the point of force is invariant throughout the force measurement operation. Equation (22) shows that the detection point displaces in the same direction as the exerted force. Thus, the external force can be measured from the displacement of the detection point.

## 4. Development of the System

### 4.1. Development of the Structure for the Vertical Direction Force Measurement in the mN Range

**Figure 7:**
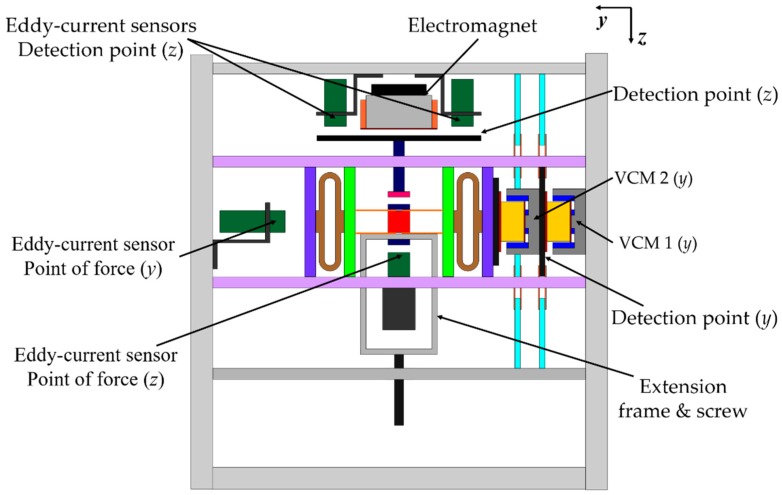
View of the developed system [23].

**Figure 8:**
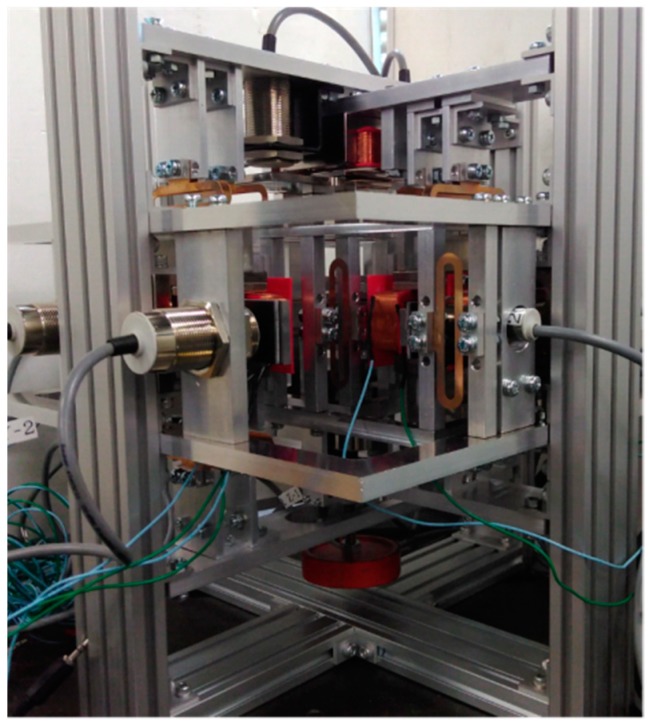
Photograph of the instrument [23].

For a vertical direction force measurement operation, an electromagnet (0.5 mm in diameter coil and 378 turns) was fixed to the top base frame, as shown in Figure 7. An iron plate was supported by leaf springs to act as the detection point. Two eddy current gap sensors were attached to the top base frame to measure the movement of the detection point. A permanent magnet was installed at the tip of the extension part of the detection point. The point of force was also suspended with leaf springs. An extension frame was connected to the point of force for applying external force. Another eddy current sensor was fixed to the bottom base frame to estimate the motion of the point of force. A photograph of the developed device is presented in Figure 8.

### 4.2. Setup for Microforce Measurement

**Figure 9:**
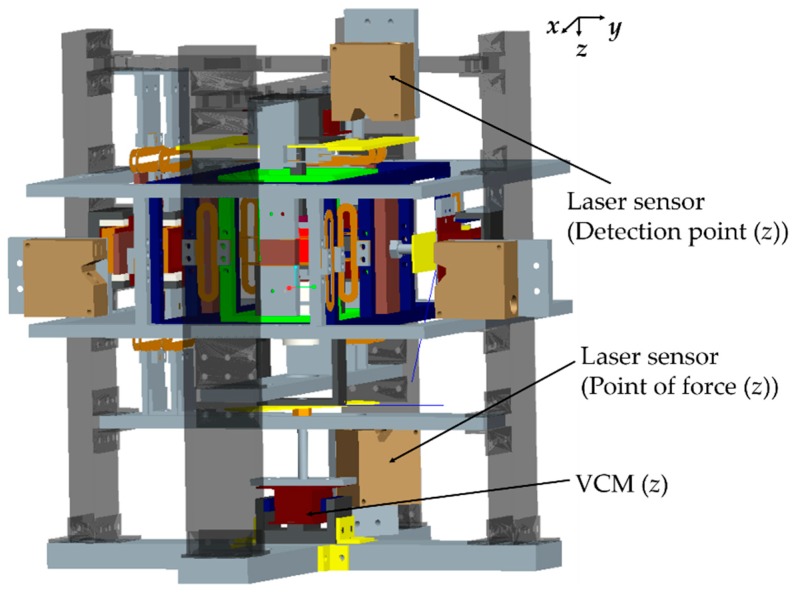
Schematic diagram of the microforce measurement setup.

**Figure 10:**
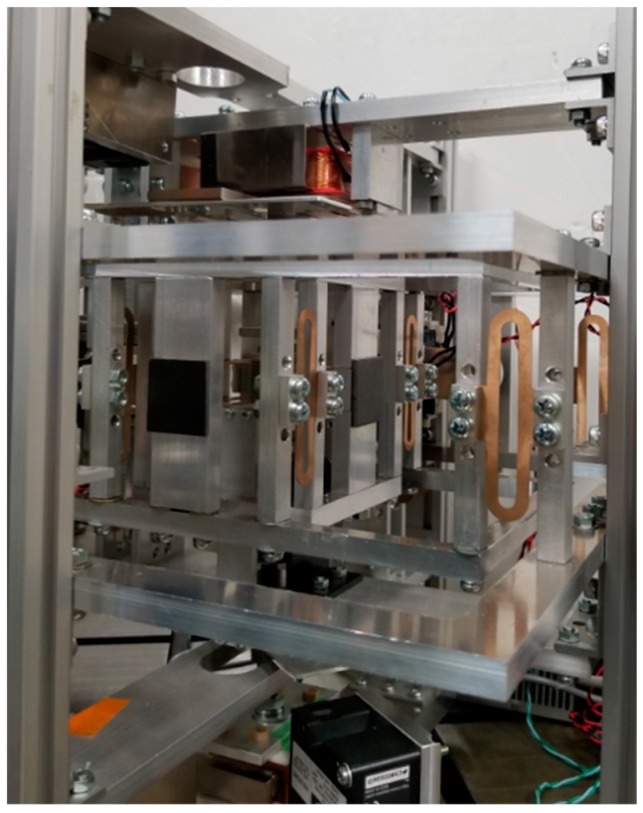
Photograph of the microforce setup.

The structure of the mN range force measurement device was modified with additional voice coil motors (VCMs) to apply microforce at the point of force in the vertical *z* and horizontal *y* directions, as shown by Figure 9. To measure displacements of the point of force and the detection point in the μm range, high-resolution laser sensors were used instead of eddy current gap sensors. A photograph of the device in the microforce measurement setup is presented in Figure 10.

## 5. Experimental Results

**Figure 11:**
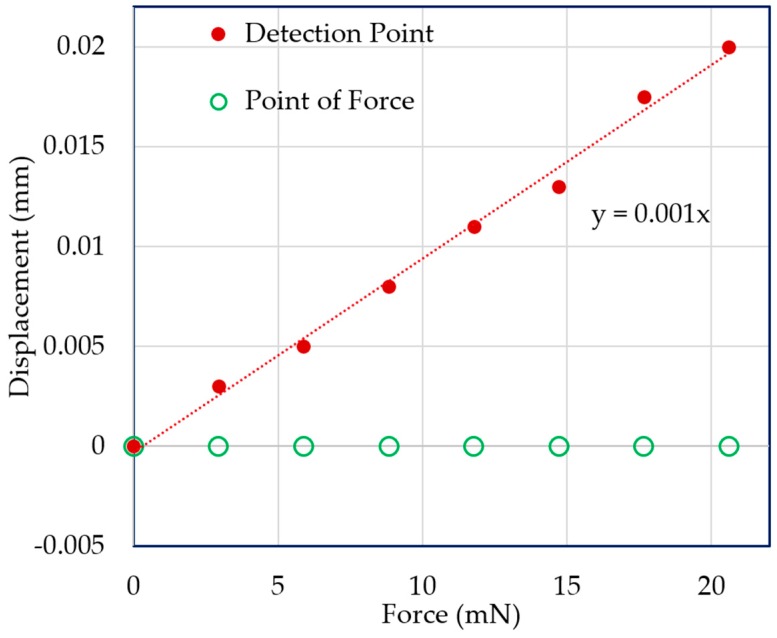
Displacements of the point of force and detection point with the addition of a unit force of 3 mN.

**Figure 12:**
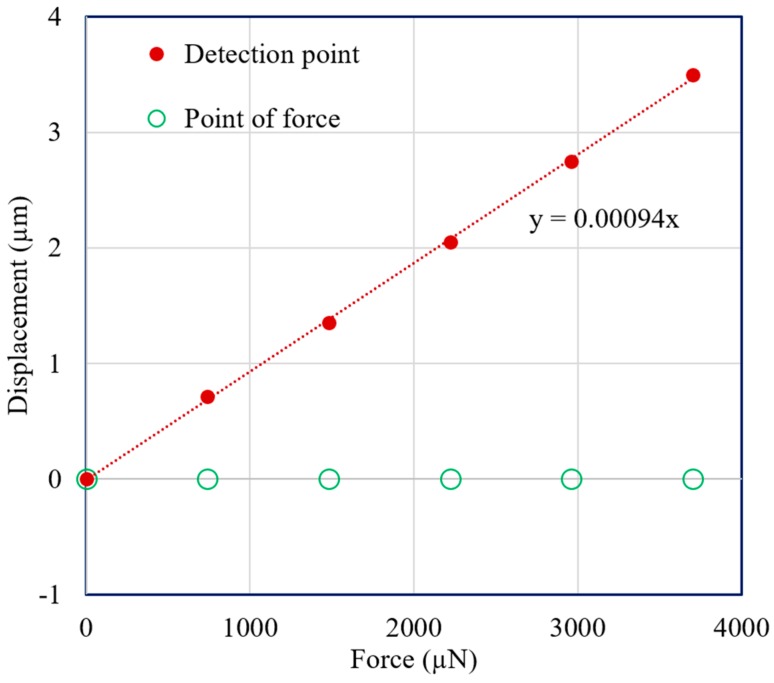
Displacements of the point of force and detection point with the addition of a unit force of 740 μN.

Vertical direction force was applied at the point of force by using the VCM instead of adding weights with the extension screw. A force measurement experiment was carried out for both the mN and μN ranges. In both cases, the displacement of the point of force and the detection point were measured with the laser sensors. Figure 11 and Figure 12 demonstrate the experimental results for 3-mN and 740-μN unit force, respectively. The displacement-force graphs shown by Figure 11 and Figure 12 present that the position of the point of force was kept invariant, and the detection point displaced linearly in the same direction as the applied force. Thus, the experimental result followed the theoretical analysis, and the external force could be measured form the displacement of the detection point. Therefore, stable zero-compliance was achieved on both the occasions of mN and μN range force measurements, and the detection point moved linearly in the direction of the exerted force to satisfy the theoretical analysis. 

**Figure 13:**
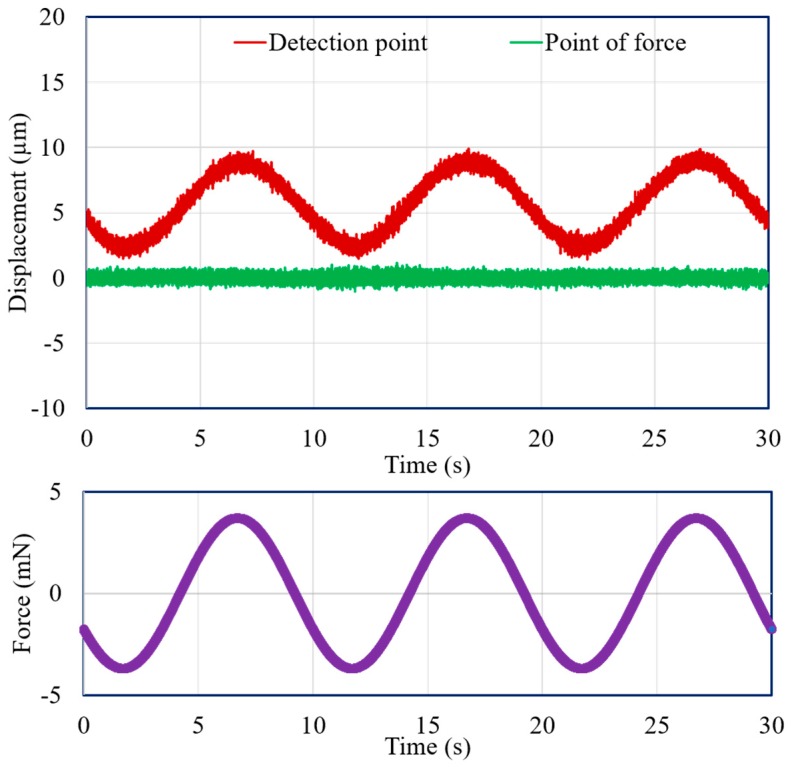
Displacements of the point of force and detection point with a dynamic force of 3.7 mN and a 0.1-Hz frequency.

**Figure 14:**
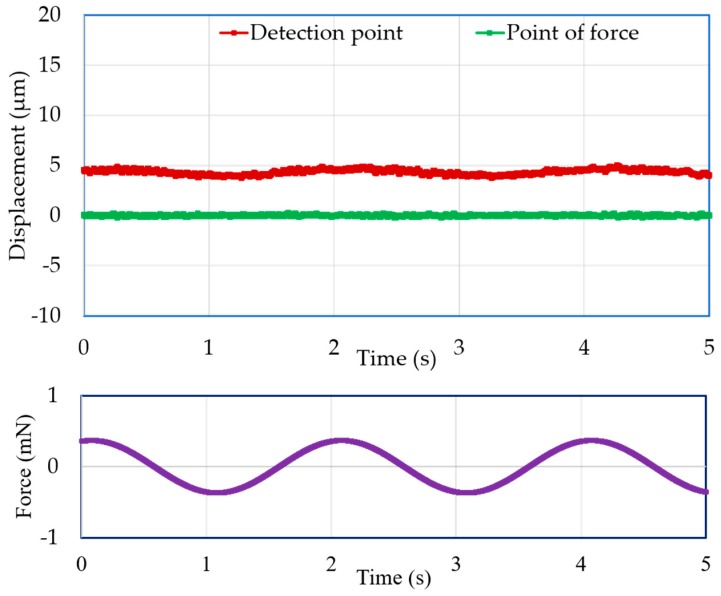
Displacements of the point of force and detection point with a dynamic force of 555 μN and a 0.5-Hz frequency.

Moreover, the experimental results for dynamic force with an amplitude of 3.7 mN and a frequency of 0.1 Hz, and with an amplitude of 555 μN and a frequency of 0.5 Hz, are presented in Figure 13 and Figure 14, respectively. Both figures demonstrate that the detection point displaced according to the applied force, and a stable zero-compliance condition was obtained at the point of force.

## 6. Conclusions

Modeling of a vertical direction force measurement operation of a three-dimensional force estimation instrument was validated by the results of force measurements for the mN and μN ranges. High-resolution laser sensors were used to measure the displacement of the point of force and the detection point in the μm range instead of eddy current gap sensors used to measure force in the mN range. A micronewton order force was applied with a VCM attached to the bottom frame. A stable zero-compliance condition at the point of force was confirmed in both the mN and μN range force measurements, and the linear movement of the detection point was ensured. In addition, force was also estimated in a dynamic condition, and the experimental result confirmed a steady zero-compliance condition at the point of force. Thus, the experimental results validated the analyses of the vertical direction force measurement both in static and dynamic conditions and in the mN and μN ranges. 

## Figures and Tables

**Table 1:** Parameters by using the Butterworth standard form.

Serial No.	$f_{n}\;[\mathrm{Hz}]$	$p_{d}^{\left(z\right)}\;[A/m]$	$p_{v}^{\left(z\right)}\;[\mathrm{As}/m]$	$q_{d}^{\left(z\right)}\;[A/m]$	$q_{v}^{\left(z\right)}\;[\mathrm{As}/m]$	$q_{I}^{\left(z\right)}\;[A/\mathrm{ms}]$
TF1	10	−768.767	13.25	263.803	−60.9846	24901.8
TF2	20	3270.36	26.5	−9157.61	76.3943	796857
TF3	30	10002.2	39.75	26466.2	610.5	6.05113 × $10^{6}$
TF4	40	19426.9	53	184125	1739.7	2.54994 × $10^{7}$
TF5	50	31544.3	66.25	571602	3662.35	7.7818 × $10^{7}$
